# Entrepreneurship programs and their underlying pedagogy in secondary education in the Netherlands

**DOI:** 10.1007/s41959-022-00078-8

**Published:** 2022-10-12

**Authors:** Sultan Göksen-Olgun, Wim Groot, Ingrid Wakkee

**Affiliations:** Business and Economics, Maastricht University, Minderbroedersberg 4-6, 6211 LK Maastricht, The Netherlands

**Keywords:** Entrepreneurship education, Design principles, Entrepreneurship pedagogy, Secondary education

## Abstract

Despite the increase in entrepreneurship initiatives and interventions, there is a poor understanding of entrepreneurship programs' principles and learning objectives in secondary education. This study provides insight into the current range of entrepreneurship programs and their underlying pedagogy in secondary education in the Netherlands. To structure the analysis, we used the 11 design principles of Baggen et al. (Entrep Educ Pedagog 5(3):347–374, 2022). Data were collected from three different sources: the Vecon Business Schools (VBS) application forms, interviews with VBS schools, and additional documents. The findings show that most schools are unfamiliar with the definitions of entrepreneurship education. Many of the principles of broad entrepreneurship education focus on personal growth and the development of an entrepreneurial mindset. These are not always recognizable in secondary schools' current offerings of entrepreneurship education. Furthermore, the pedagogies on which entrepreneurship programs are based are mostly traditional, despite literature showing that (socially) constructivist entrepreneurship programs lend themselves better to developing an entrepreneurial mindset.

**Supplementary Information:**

The online version contains supplementary material available at 10.1007/s41959-022-00078-8.

## Introduction

Technological innovation and economic globalization have increased the demand for entrepreneurs and workers with an entrepreneurial mindset (Kuratko et al., 2015; Landström et al., 2012). This development has led to a sharp increase in entrepreneurship education at all levels of education, including primary and secondary schools. Also, in the Netherlands, the supply of entrepreneurship education for young people has grown in recent decades due to, among other things, changes in the curriculum of economics education (Boot & Kolkman, 2016). The scope of entrepreneurship education, especially in primary and secondary education, has broadened from training future entrepreneurs and small business owners to personal development and developing an entrepreneurial mindset. This mindset is intended to benefit individuals and society (Bacigalupo et al., 2016). It aligns well with the ambition of the European Commission, which sees entrepreneurship as a critical competence for personal development growth (knowledge and skills), active citizenship, social inclusion, and career opportunities in the labor market (European Commission, 2006; Hägg & Kurczewska, 2021).

Previous studies have shown a positive impact of entrepreneurship education on the (personal) development of (young) students and their chances in the labor market (Hadley, 2022; Huber et al., 2014; Kim et al., 2021). This makes the provision of entrepreneurship education valuable, especially for young people. At the same time, the entrepreneurship curriculum/programs and the pedagogy behind entrepreneurship education are critical factors in achieving this impact (Hadley, 2022). Nevertheless, much of the existing literature in this area focuses on higher and vocational upper-secondary education. Secondary education, or the young entrepreneur, still receives little attention (Elert et al., 2014; Fayolle, 2018; Hägg & Kurczewska, 2021). In this study, we aim to contribute to this knowledge gap by generating insights into;

1. the entrepreneurship curriculum/programs offered in upper secondary schools in the Netherlands, by analyzing their design principles focused on broad entrepreneurship education and
2. by generating insights into the underlying pedagogy of the entrepreneurship programs offered to determine how best to teach these students.

Analyzing entrepreneurship curriculum/programs requires some agreement on the criteria used in designing entrepreneurship curriculum/programs. As a result, in this study, we use the 11 design principles developed by Baggen et al. for broad entrepreneurship education (2022). While the 11 design principles of Baggen et al. (2022) are not the only design principles available for analyzing entrepreneurship programs and their underlying pedagogy, they are the most relevant to our study (Löbler, 2006; Naia et al., 2014). These design principles can be used at different educational levels, in contrast to the other principles (Löbler, 2006; Naia et al., 2014), and focus, among other things, on the development of an entrepreneurial mindset (Bacigalupo et al., 2016). Also, the design principles provide empirical insights into the design criteria and underlying pedagogies that should be part of a curriculum/entrepreneurship program and thus can be used as a basis for comparing existing entrepreneurship programs offered in different contexts.

This research aims to analyze offerings of youth entrepreneurship education in terms of design principles in the Netherlands in order to make the underlying pedagogy transparent. This contributes to the literature and practice of two disciplines: education and entrepreneurship. We collected data to analyze entrepreneurship programs in secondary schools in the Netherlands. We interviewed teachers affiliated with the largest umbrella organization for entrepreneurship education in secondary schools, the VECON Business School (VBS). VBS member schools are spread throughout the Netherlands, include all secondary education levels, and are relatively representative of schools in the Netherlands (Appendix 1 in supplementary material).

## Literature review

### Definitions

This study contributes to the literature by examining existing entrepreneurship programs and their underlying pedagogy designed for secondary school youth entrepreneurs (ages 14–18). To discuss entrepreneurship education in different secondary schools, it is essential to come to a consensus on the definition of entrepreneurship education. Entrepreneurship has been assigned several definitions over time, making defining the term entrepreneurship education challenging (Kim et al., 2021; Garavan & O'Cinneide, 1994; Gartner, 1990; Pittaway & Cope, 2007). The best-known definitions in the literature are "enterprise education" and "entrepreneurship education." Whereby "enterprise education" is about transferring business knowledge and training individuals to start and manage new businesses, the so-called narrow approach.

On the other hand, "entrepreneurship education" is about a broad approach to entrepreneurship (Hannon, 2018) as a "key competence for personal development (knowledge and skills), active citizenship, social inclusion and career opportunities in the labor market" (European Commission, 2006; Hägg & Kurczewska, 2021). This study uses the broad term "entrepreneurship education" because we deal with secondary school students (ages 14–18). In the literature, actions of this age group about entrepreneurship are referred to as "youth entrepreneurship" (Hadley, 2022; Kim et al., 2021). Despite the increase in entrepreneurship at different levels of education, including secondary schools in other countries (Busenitz et al., 2014; Kim et al., 2021), little research has been done on this target group. The significant difference between young and adult entrepreneurs is that youth entrepreneurs are further removed from the labor market and enter the economy later than college or university students. As a result, a broad approach to entrepreneurship, with an emphasis on personal development, is more appropriate for this target group than a narrow approach, focused on training individuals to start and manage new businesses (Busenitz et al., 2014; Hadley, 2022; Kim et al., 2021).

### Learning theories and underlying pedagogies

Learning theories and their underlying pedagogies have become increasingly relevant in entrepreneurship education. This study defines pedagogy as theories and models of teaching and learning (pedagogy & didactics), similar to the studies of Kyrö (2005), Lackéus (2020). Several studies have shown that using specific pedagogical approaches influences the development of students' entrepreneurial self-efficacy, attitudes, and mindsets (La Guardia et al., 2014; Wardana et al., 2020). In addition, learning theories and their underlying pedagogy often form the basis of classifying, designing, and developing entrepreneurship education (Lackéus, 2020). Below we explain the most commonly used learning theories in entrepreneurship education and their underlying pedagogies and design principles.

#### Learning theories in entrepreneurship education

In the literature, we can divide the learning theories into three main strands (1) behaviorism, (2) cognitivism, and (3) (social) constructivism. Below we will explain these three strands. The most traditional learning theory in entrepreneurship education is (1) behaviorism. In behaviorism, learning occurs due to an observable change in behavior based on repetition and external stimuli, where positive behavior is rewarded and reinforced. The pedagogy behind this learning theory rests on a passive and instruction-based teaching approach, aiming to achieve predefined behavior (Hägg & Kurczewska, 2021; Krueger, 2007). In contrast to the general education system, this learning theory is frequently used in entrepreneurship education because entrepreneurship education revolves around changes in human behavior (Hägg & Kurczewska, 2021). Hence, this is also why the theory of planned behavior (Ajzen, 1991), which states that (behavioral) intention is the most crucial predictor of intended behavior, is often used for impact measurement within entrepreneurship education (Heuer & Kolvereid, 2014; Nabi et al., 2017).

On the other hand, more recent cognitivist learning theories (2) provide the basis of the current general education system. Cognitivism focuses on individual knowledge acquisition, based on the learner's cognition, where behavior results from the thought process (Kozlinska, 2016; Krueger, 2007; Mueller & Anderson, 2014). The pedagogy behind this learning theory relies on an individual teaching approach based on the cognitive level of the learner (Bandura, 1971; Mueller & Anderson, 2014). Cognitivism has many applications because students, especially in secondary education, learn from methods, and teachers test this through a summative assessment (e.g., tests, final exams). In the general education system, cognitivism is often mixed with behaviorism (Kozlinska, 2016). Also, in entrepreneurship education, we see examples of a mix between cognitivism and behaviorism (Kozlinska, 2016), such as the accounting and marketing modules based on knowledge acquisition.

A deepening of cognitivism is constructivism (3), in which learning is a subjective process. Within this learning theory, the learner is cognitively challenged to adopt a critical attitude toward the known and reflects on the unknown (Hägg & Kurczewska, 2021; Mueller & Anderson, 2014). Social constructivism is a component of constructivism that refers to socialized learning and the socially situated context of cognition (Hägg & Kurczewska, 2021; Krueger, 2007). In these learning theories, the responsibility for learning and the learning process rests with the learner, the teacher, and providing a learning space, acting primarily as a coach and process facilitator. We also see this in examples of experimental learning, such as problem-based learning and business games (Pech et al., 2021; Sihaloho, 2021). Researchers often see these forms of constructivism as the most appropriate form of entrepreneurship education for secondary and primary school students (Hägg & Kurczewska, 2022; Lackéus, 2020; Moberg, 2014).

#### Pedagogy within entrepreneurship education

Within the pedagogy of entrepreneurship education, three didactic models are distinguished: education "(1) about," "(2) for," and "(3) through" entrepreneurship (Aadland & Aaboen, 2018; Kozlinska, 2016; Lackéus, 2020). This didacticism about teaching entrepreneurship is one of the most widely used classifications in the pedagogy of entrepreneurship education (Aadland & Aaboen, 2018; Hägg & Kurczewska, 2021).

The most passive form of the three didactic models involves teaching "(1) about" entrepreneurship, also called the "supply model" or "the traditional model" (Kozlinska, 2016). The teacher is an expert within this form, whose main task is knowledge transfer. According to general learning theory, this form of pedagogy falls under behaviorism and cognitivism, in which individual knowledge acquisition is central (Hägg & Kurczewska, 2021; Mueller & Anderson, 2014). This form of teacher-centered pedagogy is not seen as appropriate for entrepreneurship in secondary education because secondary school students are further removed from the labor market and need an environment with room for space to work on their personal and professional development (Hadley, 2022).

Education "(2) for" entrepreneurship, also called the "demand model" (Kozlinska, 2016), focuses on activating education to teach entrepreneurial skills and mindset. The general learning theory relates to constructivism, which focuses on self-regulation in making choices and taking responsibilities (Mueller & Anderson, 2014).

The final didactic form involves education "(3) through" entrepreneurship, also called the "competence model" (Kozlinska, 2016); this consists of a combination of knowledge transfer (instrumental approach), skills learning and developing an entrepreneurial mindset (entrepreneurship method approach). Moberg (2014), Lackéus (2014), and Hadley, 2022) state that education "through" entrepreneurship has the most positive effect on entrepreneurship skills of primary and secondary school students in terms of proactivity, engagement, and enjoyment of school. Within the entrepreneurship pedagogy, we have seen a shift from behaviorism (educationally also from cognitivism) to constructivism since the 1980s (Hägg & Gabrielsson, 2019). This shift is mainly due to the increased popularity of the theories of Bruner (1996), Piaget (2000), and Vygotsky and Cole (1978), which focus on experiential learning and problem-based learning. In addition, the European Commission's (2013, p. 7) advice is to provide students with at least one practical entrepreneurship experience, based on the form "through" entrepreneurship, before they leave compulsory education (Hadley, 2022). According to general learning theory, this is a mix of constructivism and cognitive learning theory.

### Design principles in entrepreneurship education

Analyzing entrepreneurship programs and their underlying pedagogy requires agreement on design and architecture, definition, and pedagogy. Design and architecture provide a systematic basis for comparing entrepreneurship programs in different contexts. Several studies have formulated design principles or architectures for designing entrepreneurship education (Baggen et al., 2022; Fayolle & Gailly, 2008; Löbler, 2006; Naia et al., 2014). These design principles or architectures are based on the micro levels of entrepreneurship education. Students are exposed to context-specific entrepreneurship knowledge, skills, and attitudes rather than a cross-curricular embedding (Hadley, 2022). Hence, there are almost no examples in secondary education of cross-curricular methodologies to teach entrepreneurship knowledge, skills, and attitudes. However, we see in the literature that many of these micro design principles of entrepreneurship education are based on higher education or university education, except for the design principles of Baggen et al. (2022). The 11 design principles of Baggen et al. (2022) are recently designed but theoretically well-grounded in experiential learning theory (Kolb & Kolb, 2005), which fits well with (social) constructivism learning theory and broad entrepreneurship education, and thus with our target audience of young entrepreneurs, or high school students (Hadley, 2022; Lackéus, 2014). Also, the design principles allow for reflection on the current entrepreneurship program by placing the principles on a two-sided scale that can indicate the degree of presence based on the context and the target audience. Baggen et al. (2022) summarize the design principles into three categories: (1) the entrepreneurship process, (2) the task, and (3) the context and relationships. Below we will discuss each category.

#### The entrepreneurship process

The first category concerns the design principles: the method, the level of autonomy, and the room to maneuver. The method revolves around the principles on which the method within the entrepreneurship education offered is based; for example, these may be based on a narrow definition (traditional/business planning), broad definition (constructivist/personal development), or a combination of the two. The degree of autonomy is often an extension of this method, which revolves around the autonomy the student experiences over their learning and decision-making process in the value creation process. Moreover, the room to maneuver emphasizes entrepreneurship education's iterative, experimental nature (Baggen et al., 2022).

#### The task

The second category concerns the design principles: the complexity of the cases students face, the nature of the value creation process, the knowledge creation process, and the impact of the results. The complexity refers to the degree of complexity and uncertainty students are dealing with in assignments. Like the value creation process, these can be a single or multiple value creation. Because entrepreneurship in secondary education in the Netherlands is offered as part of the subject of economics, the question is whether there are also social, cultural, or other combinations of value creation in addition to economic value creation. The knowledge creation process involves innovating or transforming domains to create value, which requires creating new (domain-specific) knowledge. Furthermore, in the impact of the result, it is essential for whom the value creation process takes place, for example, peers, teachers, or external stakeholders (Baggen et al., 2022).

#### The context and relationships

The third and final category concerns the design principles: context/environment, collaboration, the role of external stakeholders, and role models. The design principle context/environment is about at what level the value creation takes place in a specific context, which can be local or international. For cooperation, the degree and type of partnership with others can vary, ranging from collaboration with peers, (un)known external parties, or heterogeneous/interdisciplinary teams. The role of external stakeholders revolves around the involvement of external stakeholders in the entrepreneurship education offered and the complexity of the entrepreneurship challenge made available by external parties. Finally, role models in the offer can vary from inspiring students to act entrepreneurially to coaching/supporting students in developing their own entrepreneurial identity (Baggen et al., 2022).

## Methods

This study uses a qualitative research design for a deeper and broader understanding and insight into the entrepreneurship programs offered and the underlying pedagogy in upper secondary schools. Bryman (2016) states that a qualitative research method focusing on semi-structured interviews is a well-known and widely used technique in the qualitative research approach. Furthermore, van Burg et al. (2022) note that qualitative research is a good fit for studying new, underexposed, or difficult-to-measure entrepreneurship phenomena. Therefore, in this study, we chose a qualitative approach, with both deductive and inductive content analysis. To this end, data are obtained through the analysis of documents of 59 schools (application forms for a VBS certificate in entrepreneurship and supplementary documents) and semi-structured interviews with 41 teachers from 36 schools across the Netherlands.

### General info on secondary education in the Netherlands

After primary education, students are enrolled in the following level groups in secondary education, depending on their test scores (1) pre-vocational secondary education (VMBO, duration of four years), (2) senior general secondary education (HAVO, duration of five years) and (3) pre-university education (VWO, duration six years). Ultimately, the (1) VMBO prepares students for secondary vocational education (MBO). Moreover, the (2) HAVO and (3) VWO courses prepare for tertiary education/higher education. Students are offered entrepreneurship in upper secondary education (age between 14 and 18 years) in the Netherlands, provided they choose it. Almost all secondary schools offering entrepreneurship education are members of the VECON Business School (VBS) umbrella organization. Every two years, the umbrella organization VBS approves whether schools may identify as "entrepreneurship/business schools" by using application forms to assess whether they offer sufficient activities to promote entrepreneurship. In addition, the umbrella organization makes certificates available to schools and students who have completed the entire entrepreneurship program. In 2022 this umbrella organization had 71 member schools in secondary education, which together provide a balanced spread in both location and levels (Appendix 1 in supplementary material).

### Data collection

For triangulation, data were collected from three different sources (Yin, 2009)—application forms from schools affiliated with the VECON Business School (VBS), (2) semi-structured interviews with teachers, and (3) supplementary documents. We distinguish two steps in collecting the data:

(1) collecting the application forms from schools affiliated with the VECON Business School (VBS); (2) preparing, conducting, and transcribing interviews; and (3) the supplementary documents. These two steps are explained below.

*Step 1* Collecting application forms from schools affiliated with the VBS

We used data from the application forms (2021–2022) that schools must complete to (re)certify as a VECON Business School (VBS). The forms include general information about the school, the profile of the (entrepreneurship) school (including mission and vision), the entrepreneurship curriculum, and any attachments. We used the application forms because, in addition to the general information and profile of the schools, they also show the content of the entrepreneurship curriculum offered. Of the 71 school members of the VBS umbrella organization, 58 schools made their application forms available for this study. Forty-one teachers from 36 schools that provide entrepreneurship education were interviewed (see Appendix 1 in supplementary material overview of schools). These interviews form the basis of our results section.

*Step 2* Preparation, conducting, and transcribing of structured interviews and the supplementary documents

### Preparation

In preparation for the interviews, the application forms, which contain information about the visions, missions, learning goals, number of students, teachers, and content of the entrepreneurship program offered, were studied. This information was used to design the guiding principles for the semi-structured interviews. This guideline was shared and submitted to experts for feedback, and the feedback received was incorporated. The procedure for the semi-structured interview included: an introduction, introductory questions (level, numbers, mission/vision, embedding curriculum, content, programs, and learning goals), the 11 design principles, and some concluding questions (student/teacher experiences, underlying pedagogy, evaluation 11 design principles). Additionally, a specific interview guideline was developed for each school. Within this particular guideline, the school was already scored according to the design principles on a three-point scale, identifying programs that are cognitivist (teaching "about" entrepreneurship), constructivist (teaching "through" entrepreneurship), or a mix of both forms (teaching "for" entrepreneurship) to gain insight into the underlying pedagogy of entrepreneurship programs (Moberg, 2014; Mueller & Anderson, 2014).

Next, the data from the application forms were used to prepare for the interviews by adding all the information from the application forms into the interview guide. The pre-completed interview guide was shared with the participating schools so that the schools could review, discuss, and possibly score the interview guide on a three-part scale in advance, along with the rest of their colleagues in the section. The results of the discussions were then brought in by the interviewee during the interview.

### Conduct interviews

In this study, we used a single sample, approaching all 71 members (schools) of the VECON Business School. In the end, 36 schools and 41 teachers participated in the semi-structured interviews. These 36 schools represent more than half of the 71 VECON Business Schools and are well distributed over the Netherlands and over the different levels (see Appendix 1 in supplementary material). The interviewees all had experience in teaching and developing entrepreneurship education. The interviews were conducted between 01-11-2021 and 01-03-2022 by COVID online. We stopped after 36 semi-structured interviews because theoretical saturation was reached.

### Transcription

Each interview lasted an average of 75 min and was recorded and transcribed verbatim. After the interview, some participating schools shared additional documents related to their mission/vision, learning goals, or teaching materials.

### Data analyses

The lead author analyzed all interview data using the MAXQDA interview coding software. Both a deductive and inductive approach was used in coding the semi-structured interviews. The deductive approach was based on the design principles of Baggen et al. (2022). Furthermore, the inductive method was based on all the data that were not directly related to the design principles. For these data, own categories and labels were formed. A three-step protocol was used to analyze the deductive and inductive approaches, similar to previous research on youth entrepreneurship, which will be explained below (Hadley, 2022). The three-step protocol:

#### Linking and creating codes

This step was very labor-intensive because we used both a deductive and inductive approach. To do this, we needed to read and reread all data to classify them by design principle and then score on a three-part scale: identifying programs that are cognitivist (teaching "about" entrepreneurship), constructivist (teaching "through" entrepreneurship), or a mix of both forms (teaching "for" entrepreneurship) to gain insight into the underlying pedagogy of entrepreneurship programs (Moberg, 2014; Mueller & Anderson, 2014). Any data we could not score, such as level, numbers, mission/vision, curriculum embedding, and learning objectives, but were necessary for context, were coded inductively.

#### Identify emerging themes, patterns, and relationships

The data were structured in deductive and inductive approaches. The deductive approach helped categorize and score schools on the presence of design principles and the range of pedagogies. Appendix 2 in supplementary material describes the scoring method for each design principle, based on a three-level scale: low/easy, hybrid, and high/complex. The inductive approach helped understand the context and underlying values (vision/mission/learning goals) of the entrepreneurship education offered.

#### Summarizing the data

After coding the interviews, tables were generated to provide input for the analyses, such as context information of the participating schools, an overview of entrepreneurship programs, an overview of scored design principles, and the underlying pedagogies of the entrepreneurship programs. Based on this, patterns and relationships were recognized, and the strengths and weaknesses of the various entrepreneurship programs and the underlying pedagogical principles were identified and quantified.

### Reliability

After coding, we analyzed the data and clustered them by design principle. The clustering of the first interview was emailed to Baggen, one of the designers of the 11 design principles (Baggen et al., 2022), to receive feedback on the interpretation of the interview. The input included interpreting the design principles and clustering the design principles by the level of presence (low versus high). This feedback was processed and used in processing the other interviews. In some cases, the authors expressed doubts about the classification. These cases were discussed with all authors and six colleagues from the Centre for Applied Research on Economics & Management (CAREM) knowledge center in three sessions ranging from 50 to 80 min until a consensus was reached.

## Findings

We have divided the description of the results into two sections. In the first part, we describe the entrepreneurship curriculum/programs offered in upper secondary schools in the Netherlands by analyzing them on design principles focused on broad entrepreneurship education (deductive).

In the second part, we discuss the school context, including the mission, vision, and learning goals, by generating insights into the underlying pedagogy of the entrepreneurship programs offered to determine how best to teach these students (inductive).

### The 11 design principles

In this section, the 11 design principles of Baggen et al. (2022) are used to illuminate the existing provision of entrepreneurship education within secondary schools, using the findings summarized in Table 1.

**Table 1:** The results

Design principle (Baggen et al., 2022)	Example citations	Low(er) levels of complexity and uncertainty	Hybrid	High(er) levels of complexity and uncertainty
1. *The entrepreneurial process*				
The principles of the method (causation versus effectuation)	‘Yes, it is variable. [..] The business plan is more like effectuation, we do, of course, provide a kind of format for students to work within. So yeah, it's a bit of both sides.’	Causation-oriented education: Of the 36 schools interviewed, 17 offer programs within entrepreneurship education offered that fall entirely under causation-oriented education, with predetermined goals, such as accounting, marketing, and other business programs/assignments	Combination of causal and effect-oriented education: Within the entrepreneurship education offered, all schools offer some programs that either focus on setting up a (fictional) business or writing a business plan/business canvas model (see Table 1)	
Level of autonomy (learning/decision-making process)	‘They do get the guidance, but they get a lot of freedom. And um, yes, the advice is mainly to adjust if we see that it goes the wrong way.’	The degree of autonomy depends on the entrepreneurship program offered. Entrepreneurship programs that fall entirely under causally oriented education, with pre-formulated goals, such as accounting, marketing, and other business programs/assignments, are offered in a teacher-driven way, just like traditional economics, and business economics subjects’ degree of autonomy is low	Within the entrepreneurship programs focused on setting up a business, writing a business plan, or designing a business canvas model, students often get more space to work on their business ideas independently or in groups	
Room to maneuver (stimulates out-of-the-box thinking, experimenting and prototyping)	‘Of course, you also try to support as much as possible, allow mistakes, and learn from them. But with the profile piece, of course, there will be an assessment moment, and with a diploma in accounting, there will also be an assessment moment.’	In most schools (30 out of 36 interviewed), there is trial and error and reflection in a safe environment without time pressure. In some cases, students are only required to design the business plan, making it complicated to conduct multiple iteration/experimentation rounds	There are also schools, 14 out of 36 interviewed indicate to do some form of reflection using coaching during the project or at the end of the project (how they experienced it) using Personal Development Plan conversations (POP-conversations)	
2. *The task*				
Complexity of the cases students face (key for value creation)	‘No, the complexity was really very low. Um, I sometimes thought it was too low. At the same time, this was really challenging enough […].’	The assignments in the causal programs are not complex but theoretical For example, 32 of the 36 schools interviewed indicated that they usually involve relatively simple business ideas that require little innovation or financial resources (e.g., designing packaging for an existing product or combining existing products, such as a pillow with a suction cup for travel)	There are also schools (15 of the 36 interviewed) that offer at least one or sometimes even a few projects that challenge students to find solutions to complex problems Innovation does not play a central role but is a positive external effect	
Nature of the value creation process (i.e., multiple value creation)	‘It's mainly, um, economical with a bit of social um, broadening into the social.’’	Most schools (26 of the 36 interviewed) focus on economic value creation. If students themselves come up with other forms of value creation, this is encouraged but is not explicitly included as a requirement in assignments	In total, 24 of the 36 schools interviewed indicated that they do create value indirectly but are not consciously working on it. The examples given are; selling stuff through mini-companies that benefit the neighborhood and the school or donating money that students earn from their mini-companies to charities	There are also schools (6 out of 26 interviewed) that have projects in their entrepreneurship program that focuses on multiple value creation, such as economic, social, environmental, and cultural value creation (e.g., projects focused on Global Goals, Day for Change, Social Innovation Relay and Guest Lectures)
Knowledge creation process (innovative)	‘It still remains simple, […] research is being done into it, but to elaborate on that and actually do it, we haven't come that far yet. ‘	In almost all of the schools interviewed (except two), the process of value creation is driven by intuition and curiosity; domain-specific knowledge is often not needed. Students have to put business economics theories into practice		In a few schools (2 out of 36 interviewed), students have to use new (domain-specific) knowledge in one or a few projects (e.g., Student Company and Social Innovation Relay from Young Entrepreneurship and the Global Goals project)
Impact of the result (peers/teachers versus stakeholders)	‘The value created is mainly visible in the grade for the profile piece, the certificate. But a portfolio would be nice, of course.’	In total, 35 of the 36 schools indicated that the outcomes of the entrepreneurship programs offered include outcomes for the student (e.g., entrepreneurship diploma/certificate, entrepreneurship skills, and connection to further education) and for the teacher (e.g., grade/assessment)	A few schools (13 out of 36 interviewed) indicate that they offer projects in which students create value on a local level, such as organizing activities for a retirement home or organizing a market for the neighborhood	
3. *The context and relationships*				
Context/ environment (local/regional, to (inter)national or systemic levels)	Citation 1: ‘[…] to let students, […], get in touch with entrepreneurship, it is still easiest to do that close by for them.’	The value creation process occurs almost in all schools (33 out of 36 interviewed), mainly at the local/regional level. Students visit and receive guest lectures from local/national companies and entrepreneurs. Students also launch products and services close to their frame of reference	A few schools (4 out of 36 interviewed) offer an international project, such as a study trip to The Gambia or international internship opportunities. These are often exceptions and are offered for a small selection of students	
Cooperation (individual/peers versus heterogeneous teams)	‘Accounting is individual. Reinvesting is together […]project is also together. Um… Profile work […] is also in pairs […] and individually. So they also work together a lot, yes.’	In causality-based programs, students often work independently. Within these programs, collaboration with other students is not necessarily necessary	Also, these schools (34 of 36 schools interviewed) offer projects (student-companies) in which students do work in groups (often in groups of two to six students), but not across grade levels or classes	A few schools (8 of the 36 schools interviewed) offer projects in collaboration with further education; here, students work across levels and classes
Role of external stakeholders (complexity of the entrepreneurial challenge)	Citation 1: ‘Um, that too is ad hoc. And um, yeah… I think it's low intensity. It's sometimes just for one lesson.’	Most schools (31 out of 36 interviewed) indicated that in causal programs, students often work independently, and the role of external stakeholders is minor		Some schools (8 out of 36 interviewed) have built a network with different schools, municipalities, companies, and institutions where the intensity is high. This network benefits the schools in organizing company visits, guest lectures, and developing projects in cooperation with secondary schools and the municipality. The intensity is high, often once every two weeks or once a month
Role models (inspire versus developing entrepreneurial identities)	‘For inspiration. […], an entrepreneur often tells us: how do you come up with a business? ‘	Almost all schools indicate (34 out of 36 interviewed) that role models are used mainly to inspire students in entrepreneurship programs	A few schools (9 out of 36 schools interviewed) indicate that they also use students and teachers from further education or entrepreneurs to support or coach/mentor students during the projects. There is no direct work on identity formation; this is often done separately from the entrepreneurship program through career counseling	

The model is based on broad entrepreneurship education and is divided into three categories, namely: (1) the entrepreneurship process, (2) the task, and (3) the context and relationships. Below we will explain each category and describe the results of the 36 schools interviewed online by category.

#### The entrepreneurial process

This category includes the design principles: method, degree of autonomy, and room for maneuver. Entrepreneurship education consists of various programs, both purchased and self-designed (see Appendix 3 in supplementary material). Most schools indicate that they offer one or more traditional entrepreneurship programs, which correspond to education "about" entrepreneurship. Traditionally focused entrepreneurship programs are teacher-centered, emphasizing factual knowledge, such as accounting and marketing. According to the literature, traditionally oriented programs, which emphasize knowledge transfer, contribute less to students' entrepreneurial mindset than constructively oriented education (Moberg, 2014). For example, more than 17 of the 36 schools surveyed purchase traditional entrepreneurship programs and regular (professional) economics education, often requiring students to contribute financially.

Also, almost all schools offer constructivism-oriented education less often than traditional programs. Constructivism-oriented education corresponds to education "for" and "through" entrepreneurship. The programs under this heading are often mini-companies with business plans, projects with further education, or simulations and games. However, we did not encounter schools that offered proportionally more or fully constructivism-oriented entrepreneurship programs.

We notice the same in the degree of autonomy. Schools offer entrepreneurship programs that are traditional-oriented. These programs are teacher-driven, with students' low degree of autonomy. Similarly, in programs that schools describe as effectuation-focused education, corresponding to teaching "for" and "through" entrepreneurship, we see primarily teaching-centered education, where teachers often set the frameworks and learning objectives. Within these frameworks, students have space and autonomy to do exercises and develop skills, which usually amounts to students-companies or writing business plans. Examples of learner-centeredness and learning-centeredness we hardly encountered in secondary school programs. Finally, at most schools (30 of the 36 interviewed), we see trial and error and reflection in a safe environment without time pressure. In some cases, students only have to design the business plan, making multiple rounds of iteration/experimentation difficult.

#### The task

This category contains the design principles: the complexity of the issues students face, nature of the value creation process, knowledge creation process, and impact of the outcome. In terms of complexity, we see that assignments in the traditional entrepreneurship programs are often not complex and primarily theoretical. In contrast, studies in traditional-oriented entrepreneurship programs vary in content, duration, and complexity. For example, 32 of the 36 schools surveyed indicated that these are usually simple business ideas requiring little innovation or financial resources (e.g., designing packaging for an existing product or combining existing products, such as a pillow with a suction cup for travel). In these assignments, value creation was primarily driven by the intuition and curiosity of the students. There were also schools (6 out of 36 schools) that did offer one or a few projects that involved working on complex business ideas. However, these ideas did not need to be developed, and innovation often did not play a central role. Still, it was seen as a positive external effect, so domain-specific knowledge was often unnecessary.

In the area of (multiple) value creation, we see that most schools are not consciously and explicitly working on this (24 of 36 schools) and focus mainly on economic value creation (26 of 36 schools). Schools indicated that if students themselves came up with other forms of value creation, this was encouraged but not explicitly included as a requirement in the assignment(s). However, there are several frontrunners in terms of schools (6 of 26 schools) that have projects in their entrepreneurship program that do focus on multiple value creation, such as economic, social, environmental, and cultural value creation (e.g., projects focused on Global Goals, Day for Change, Social Innovation Relay, and Guest Lectures). In almost all schools (35 out of 36 schools) entrepreneurship programs offered outcomes primarily for the student or teachers themselves. Only a few schools (13 of the 36 interviewed) indicated that they provide projects in which students create value locally, such as organizing activities for a retirement home or a market for the neighborhood.

#### Context and relationships

This category includes the design principles: context/environment, collaboration, the role of external stakeholders, and role models.

The value creation process occurs at almost all schools (33 out of 36 interviewees), mainly at the local/regional level. Students visit and receive guest lectures from local/national companies and entrepreneurs. Students also launch products and services close to their frame of reference. The same frontrunners of schools (4 out of 36 interviewees) occasionally offer international projects. Furthermore, in terms of collaboration, we often see students working individually within traditional programs, where collaboration with other students is not necessary. The same is true for collaboration with external stakeholders, which is low. There is collaboration within projects (student companies) with fellow students (34 of the 36 schools interviewed), but not a class or cross-class collaboration. External coaches (e.g., entrepreneurs, guest lecturers, university students/teachers, companies/clients) are occasionally involved in the learning process. The intensity is often low, a few weeks before a project and often after reflection. Again, we see the same frontrunners in schools (8 of the 36 schools interviewed) that offer projects in cooperation with further education. Here students do work across levels and classes. These schools are often part of a network with different schools, municipalities, companies, and institutions where the intensity of collaboration is high. This network benefits the schools in organizing company visits, guest lectures, and developing projects in cooperation with secondary schools and the municipality.

Finally, almost all schools indicated (34 out of 36 interviewees) that role models are mainly used to inspire students in entrepreneurship programs. A few schools (precursors) (9 out of 36 interviewed) also use students and teachers from further education or entrepreneurs to support or coach/mentor students during the projects. There is no direct work on identity formation; this is often done separately from the entrepreneurship program through career counseling.

### The context

This section clarifies the context of entrepreneurship education offerings in secondary education schools. Based on the inductive approach of the interviews, we have endorsed ten concepts to describe the context, which we will explain below.

#### Definition of entrepreneurship education

Almost all of the schools interviewed, with only a few exceptions, had not thought about the definition of entrepreneurship they use within their entrepreneurship education. For example, one teacher replied, "Uhmm definition…., well that, that's just the practical interpretation of the subject…".

#### Vision and mission for entrepreneurship education

Our study shows that only seven of the 36 schools had formulated a mission or vision for entrepreneurship education.

#### Learning objectives in entrepreneurship education

Only 10 of the 36 schools interviewed had formulated overarching learning goals for entrepreneurship education. Most schools had given it little or no thought. One teacher says, "There is still profit to be made there."

#### Vertical coherence in the curriculum

The lack of a mission and vision and the absence of overarching learning goals contribute to the fragmentation of entrepreneurship education. Schools cannot test their purchased or self-designed programs against their mission, vision, or overarching learning goals, making it impossible to create vertical coherence in the curriculum. For example, 24 of the 36 schools interviewed offer entrepreneurship in the first years of secondary education. Regardless of the broad offerings, these schools lack vision and overarching learning goals, making vertical coherence in the curriculum challenging to find. Teachers indicate that they would like to work on vertical coherence in the curriculum for entrepreneurship programs in upper and lower grades in the future but have not yet reached that point.

#### Curriculum

Entrepreneurship is (in)directly offered to all students in the upper school who choose (business) economics profiles. In addition, schools are free to provide extracurricular entrepreneurship programs, which can be expressed in an extra hour per week of entrepreneurship programs, up to one full day of additional entrepreneurship (20/80 learning schools). Extracurricular components are not mandatory; students can voluntarily choose, them.

Most schools indicated that they offer extracurricular entrepreneurship programs for one or two additional hours per week. In addition, some schools have allocated more than a half or full day per week to teaching entrepreneurship. These are schools affiliated with the International Business College (IBC), which uses a 20/80 concept; all subjects are offered in four days, leaving a whole day to provide entrepreneurship education. Also included were schools that offered additional entrepreneurship classes in the regular hours of the (business) economics subjects. See Appendix 1 in supplementary material for an overview of the schools.

#### Types of programs

The traditional form, usually teacher-centered, is frequently used in entrepreneurship education (17 of the 36 schools), such as accounting and marketing modules. The teacher is seen as an expert within these programs to transfer knowledge. As in the regular subjects, these programs are offered through ready-made methods and are tested with an exam, making it easy for teachers to embed them in the regular program (Fiet, 2001a, b 1,2). In addition, schools also offer programs that fall under the constructivist learning theory and connect to learning "for" and "through" entrepreneurship, such as "mini-companies." However, these programs are still often offered traditionally. For example, one teacher describes this as follows: "The main lines are teacher-driven. They have some leeway, but you still have to meet your attainment targets."

Compared to the traditional variety, constructive assignments that are partly traditionally offered are still a step in the right direction. Constructive assignments, which are learner-centered, we did not encounter.

#### Resources

More than half of the teachers interviewed indicated they have sufficient autonomy but not sufficient resources in designing and delivering entrepreneurship education. They are often given an extra hour, but teachers indicate that designing and delivering entrepreneurship education is more complex and challenging than a standard (business) economics lesson. The lack of resources often manifests itself in development time, so teachers often choose ready-made methods based on traditional teaching because they are easy to implement and require little investment (Fiet, 2001a, b). Comprehensive programs or assignments, which are more challenging in terms of pedagogy (constructivist) and require more collaboration with the environment, and therefore more effort and background work on the part of the teacher, are less often implemented, as they quickly manifest themselves in overtime (23 out of 41 experience overtime) (Fiet, 2001a, b; Ruskovaara & Pihkala, 2013).

#### Impacts

Teachers indicated in the interviews that the entrepreneurship education offered was mainly aimed at teaching entrepreneurship skills, which students could use during their career or on the labor market. Nevertheless, these entrepreneurship skills were hardly measured or monitored. For example, 24 of the 36 schools interviewed indicated that they had not set (overarching) learning goals for their students. Moreover, only four interviewed schools show that they measure and monitor students' skills with rubrics. The remaining schools indicate that they estimate the development of skills (13 of the interviewed schools) or do nothing with it (yet) (19 of the interviewed schools).

#### Pedagogy, learning activities

According to almost all teachers, entrepreneurship education requires a different pedagogy and learning activities than regular (business) economics subjects. The described pedagogy within entrepreneurship education is mainly about coaching students, focusing on learning/developing (entrepreneurship) skills, which is in line with pedagogies such as learning "for" and "through" entrepreneurship. This pedagogy is often applied in programs where students have to set up their own (fictitious) mini-companies. Teachers explicitly indicate in these assignments that students are given extra space and act as coaches instead of teachers. Most teachers suggest that they sometimes experience this as challenging because they notice that students are sometimes not intrinsically motivated or that the school's time and resources for this type of project are often limited. Therefore they often have to motivate and adjust students in terms of the time and complexity of their ideas.

#### Teachers of entrepreneurship

In the Netherlands, entrepreneurship education in secondary schools is a part of (business) economics. Therefore, the responsibility for entrepreneurship education lies with economics teachers, regardless of whether they have an affinity with entrepreneurship. Of the 41 teachers surveyed, 17 indicated that they had no affinity for entrepreneurship. Moreover, 38 of the 41 teachers surveyed indicated that entrepreneurship education requires a different pedagogy than regular subjects such as economics and business economics. Despite this, none of the economics teacher educators surveyed were offered additional pedagogy training on entrepreneurship education. In the literature, there is an argument for teacher training programs that support a teacher during the training by working on the teacher's self-efficacy, that they can carry out entrepreneurship education and know the subject relatively well (Ruskovaara & Pihkala, 2015).

Consequently, 13 of the 41 teachers indicated a need for in-service training in teaching entrepreneurship. The remaining teachers suggested that after years of experience, they no longer need this. Finally, almost all teachers indicated that they have sufficient autonomy and insufficient resources in designing and delivering entrepreneurship education. The lack of resources often manifests itself in development time, so teachers often choose ready-made methods based on traditional teaching because they are easy to implement and require little investment (Fiet, 2001a, b). Comprehensive programs or assignments, which are more challenging in terms of pedagogy (constructivist) and require more collaboration with the environment, and therefore more effort and background work on the part of the teacher, are less often implemented, as this quickly manifests itself in overtime (23 out of 41 experience overtime) (Fiet, 2001a, b; Ruskovaara & Pihkala, 2015).

## Discussion and conclusion

This study examined the extent to which the design principles, defined for broad entrepreneurship education, are present in the current supply of entrepreneurship in the upper grades of Dutch secondary education. We also sought to generate insights into the entrepreneurship programs' underlying pedagogy to determine how best to teach students in upper secondary education. To this end, in addition to examining the presence of the 11 design principles, we also examined the context of schools, including mission, vision, learning goals, and underlying pedagogy. The results indicated that there is still much to be gained in secondary education because the 11 design principles are still minimally integrated into the current supply of entrepreneurship in upper secondary education. This is also reflected in the underlying pedagogy of entrepreneurship education on offer. This pedagogy is still mainly traditional, while the literature recommends constructivist entrepreneurship education for this young target group (Hägg & Kurczewska, 2022; Lackéus, 2020; Moberg, 2014).

Our findings highlight actions to improve the entrepreneurship education offered by: (1) organizing and offering entrepreneurship education more consciously, with attention to having a vision, learning goals, and learning activities; (2) improvements in embedding the design principles for broad-based entrepreneurship education in the current provision of entrepreneurship; (3) making learning outcomes in terms of knowledge, skills, and competencies transparent within the entrepreneurship education offered; (4) creating time and resources for teachers who teach entrepreneurship to, for example, improve their skills through education and training. We elaborate on these points below.

### Organize and deliver entrepreneurship education more deliberately, focusing on developing a vision, learning goals, and learning activities

A more deliberate organization and provision of entrepreneurship education, focusing on having a vision, goals, and learning activities, is essential for the quality of entrepreneurship education (Baggen et al., 2022; Biggs, 1996). Nevertheless, most schools have given little or no thought to their vision, goals, and learning activities (pedagogy) in the design and the reflection of the entrepreneurship education offered. In academia, we also see insufficient attention to this (Kamovich & Foss, 2017), while more evidence on this is essential for the quality of entrepreneurship education.

### Improve embedding the design principles defined for broad entrepreneurship education into current entrepreneurship offerings

We conclude that the design principles (Baggen et al., 2022) are hardly visible in upper secondary schools' current supply of entrepreneurship education. The offerings primarily consist of programs traditionally offered with knowledge transfer in mind and include accounting modules, marketing classes, and developing business plans. This finding is consistent with previous literature (Bennett, 2006; Solomon, 2007), while scholars in the field of entrepreneurship education generally agree that traditional programs and pedagogy alone are not sufficient to trigger entrepreneurial thinking and action (Hägg & Kurczewska, 2021; Joensuu-Salo et al., 2021; Maxwell et al., 2018). Furthermore, constructivist (teaching "through" entrepreneurship) delivery, especially in secondary schools, lends itself best to teaching entrepreneurship and developing an entrepreneurial mindset (Hägg & Kurczewska, 2020; Krueger, 2007; Moberg, 2014). Interestingly, schools that offer constructivist education, such as mini-companies, often stick to relatively simple local business ideas that require little innovation, financial resources, and commitment. Therefore, it is essential to continue reflecting on constructivist education. In addition, it is crucial that students, especially within broad entrepreneurship education, are encouraged to create value for others, which can be economic, cultural, social, or environmental (Baggen et al., 2022; Obrecht, 2016).

### Clarify learning outcomes regarding knowledge, skills, and competencies in entrepreneurship education

Scholars have been trying to develop insights into how entrepreneurial attitudes and skills emerge and can be developed for some time (Fayolle et al., 2014; Rosique-Blasco et al., 2016). One crucial insight is that entrepreneurship can be taught (Mwasalwiba, 2010) and that a broad mix of skills is more important for successful entrepreneurship than the highest possible degree in formative education (Elert et al., 2014). Nevertheless, from the interviews, we see that teachers are not consciously working on this, making it challenging to understand the results of entrepreneurship education in developing an entrepreneurial mindset. The reasons given by the interviewed teachers for this vary from the lack of national or international curricula/objectives for entrepreneurship education to the lack of knowledge, skills, and strong tools to do this most efficiently. Another point of interest is the learning outcomes in knowledge, skills, and competencies, which are not or hardly made insightful in the entrepreneurship education offered. In terms of future research, it would be helpful to expand on the current findings by examining what teachers need to identify the learning outcomes of the entrepreneurship education offered.

### Create time and resources for teachers who teach entrepreneurship

The final area of concern relates to teachers' time and resources to design, deliver, and reflect on entrepreneurship education. Our findings show that the (economics) teachers, who now primarily provide entrepreneurship education, are generally not explicitly educated or trained in the field of entrepreneurship and, therefore, may not have the relevant knowledge, attitudes, skills, and competencies needed to teach entrepreneurship (Gibb, 2011; Joensuu-Salo et al., 2021). This limited base may be especially problematic because many teachers report that they are often given too few hours to set up, prepare, and teach entrepreneurship (Fiet, 2001b; Joensuu-Salo et al., 2021). As a result, teachers have to use regular subjects when developing and teaching entrepreneurship (Fejes et al., 2019). Therefore further research into what teachers need and how this can be embedded in the current education system and teacher training programs is desirable.

The current study is an initial exploration of these issues. This exploration was conducted using the 11 design principles of Baggen et al. (2022) and one of the design possibilities or tools that can be used to design broad entrepreneurship education. Nevertheless, several tools or design principles can be used to create entrepreneurship education depending on missions, vision, and goals. However, in our study, we saw hardly any entrepreneurship programs that were based on design tools, principles, or methodologies. Therefore, the 11 design principles of Baggen et al. (2022) give a design opportunity or tool that can be used in designing broad-based entrepreneurship programs. Still, it is certainly not the only design opportunity or tool. Therefore, we advocate the use of design tools, principles, or methodology, in designing an entrepreneurship program in the first place. Moreover, in the second place, further research on entrepreneurship programs and the underlying didactics sheds light on learning outcomes and, consequently, on the continuous learning lines in entrepreneurship education. Further research in this important area is needed.

### Limitations

For this research, we mainly used the VECON Business schools, as this is the largest umbrella organization for entrepreneurship in secondary education in the Netherlands. In addition, the VECON Business schools are well spread across the Netherlands and include all secondary education levels in the Netherlands (Appendix 1 in supplementary material). Nevertheless, it remains interesting to do further research on the other (smaller) umbrella organizations in the future to increase the insights. Another issue was that the schools had no curricula/attainment targets or standards to focus on regarding entrepreneurship education. The lack of these curricula/endorsements or standards gave schools room to give their interpretation of entrepreneurship, which manifested in a great diversity in the provision of entrepreneurship education in secondary schools. Providing clarity in the provision through national curricula/standards/vision would help to straighten out the differences between schools and thus could positively contribute to entrepreneurship in secondary education.

## Supplementary Information

Below is the link to the electronic supplementary material.

Supplementary file1 (DOCX 41 kb)

Supplementary file2 (DOCX 24 kb)

Supplementary file3 (DOCX 34 kb)
