# Supplementary material for: Entrepreneurship programs and their underlying pedagogy in secondary education in the Netherlands
Source: Entrep Educ. 2022 Oct 12;5(3):261–87. doi: 10.1007/s41959-022-00078-8 (PMC13295173; doi:10.1007/s41959-022-00078-8)
Supplement: Supplementary file 1 — Supplementary file1 (DOCX 41 kb) [file 41959_2022_78_MOESM1_ESM.docx]

Appendix 1. Overview of schools, curriculum, province, level, teachers, students, missions & visions, and learning objectives.

| Schools | Curriculum* | Province | Level* | Interviewed teachers | Students | Documents | Engaged teachers | Mission and vision * | Learning objectives * |  |
| --- | --- | --- | --- | --- | --- | --- | --- | --- | --- | --- |
| VBS 1 | R & E1 | Overijssel | 2&3 | 1 | 205 | 2 | 2 | present | present |  |
| VBS 2 | D1 | Zuid-Holland | 2 | 1 | 48 | 1 | 1 | present | present |  |
| VBS 3 | R & E1 | Noord-Holland | 3 | 3 | 50 | 1 | 3 | not present | not present |  |
| VBS 4 | D2 & E2 | Noord-Brabant | 2&3 | - | 37 | 1 | 2 | not present | - |  |
| VBS 5 | R & E3 | Noord-Holland | 2&3 | - | 92 | 1 | 3 | - | - |  |
| VBS 6 | R (?) | Zuid-Holland | 2 | - | 42 | 1 | 2 | not present | not present |  |
| VBS 7 | R & E1 | Zuid-Holland | 2&3 | 1 | - | 1 | 1 | not present | not present |  |
| VBS 8 | D2 | Zuid-Holland | 2 | 1 | 48 | 5 | 4 | not present | present |  |
| VBS 9 | R & E1 | Utrecht | 2 | 2 | 50 | 1 | 3 | not present | not present |  |
| VBS 10 | EX10 | Zuid-Holland | 1 | - | 182 | 1 | 4 | - | - |  |
| VBS 11 | R | Gelderland | 1&2&3 | - | 243 | 1 | 4 | - | - |  |
| VBS 12 | D2 | Utrecht | 1&2 | 1 | 48 | 2 | 4 | not present | not present |  |
| VBS 13 | R | Noord-Brabant | 2&3 | - | - | 1 | 2 | - | - |  |
| VBS 14 | R & E1 | Overijssel | 1&2&3 | 1 | 40 | 1 | 3 | not present | not present |  |
| VBS 15 | R | Limburg | 2&3 | - | 60 | 1 | 3 | - | - |  |
| VBS 16 | R & E1 | Zuid-Holland | 2&3 | 1 | 155 | 2 | 3 | not present | not present |  |
| VBS 17 | R | Noord-Holland | 2 | - | 110 | 1 | 5 | - | - |  |
| VBS 18 | E2 | Zuid-Holland | 1 | 1 | 125 | 2 | 3 | present | not present |  |
| VBS 19 | R & E1 | Noord-Holland | 2&3 | 1 | 167 | 1 | 4 | present | not present |  |
| VBS 20 | R | Noord-Holland | 2&3 | 2 | 165 | 1 | 3 | not present | not present |  |
| VBS 21 | R | Zuid-Holland | 1 | - | 121 | 2 | 12 | - | - |  |
| VBS 22 | R & E1 | Noord-Holland | 2&3 | 1 | 255 | 1 | 9 | not present | not present |  |
| VBS 23 | R & E2 | Zuid-Holland | 2&3 | 1 | 350 | 1 | 7 | not present | not present |  |
| VBS 24 | EX40 | Utrecht | 2&3 | 1 | 30 | 1 | 2 | not present | not present |  |
| VBS 25 | E2 | Gelderland | 2&3 | - | - | 1 | 7 | - | - |  |
| VBS 26 | R & E1 | Groningen | 1&2&3 | 1 | 125 | 2 | 4 | present | present |  |
| VBS 27 | R | Zuid-Holland | 1 | - | 78 | 1 | 3 | - | - |  |
| VBS 28 | E2 (?) | Zuid-Holland | 2&3 | 1 | 80 | 1 | 3 | not present | not present |  |
| VBS 29 | R & P | Limburg | 1&2 | 1 | 165 | 1 | 4 | not present | present |  |
| VBS 30 | E1 | Limburg | 3 | 1 | 425 | 1 | 3 | not present | not present |  |
| VBS 31 | R | Noord-Holland | 1 | - | 200 | 1 | 4 | - | - |  |
| VBS 32 | E2 | Gelderland | 2 | 1 | 45 | 9 | 4 | not present | not present |  |
| VBS 33 | E1 | Drenthe | 1 | 1 | 120 | 1 | 10 | not present | not present |  |
| VBS 34 | D1 | Zuid-Holland | 1 | 1 | 38 | 1 | 2 | present | present |  |
| VBS 35 | E2 | Noord-Holland | 2 | 1 | 70 | 1 | 2 | not present | not present |  |
| VBS 36 | E2 | Noord-Brabant | 2&3 | 1 | 270 | 1 | 5 | not present | not present |  |
| VBS 37 | R | Zuid-Holland | 1 | 1 | 200 | 1 | 4 | present | not present |  |
| VBS 38 | R & E1 | Noord-Holland | 2 | 1 | 80 | 1 | 4 | present | not present |  |
| VBS 39 | R | Friesland | 2&3 | 1 | 10 | 1 | 4 | not present | not present |  |
| VBS 40 | R | Gelderland | 1 | - | - | 1 | 2 | - | - |  |
| VBS 41 | R (?) | Noord-Holland | 2&3 | - | 175 | 1 | 3 | - | - |  |
| VBS 42 | R & E2 | Noord-Brabant | 2&3 | 2 | - | 1 | 5 | not present | not present |  |
| VBS 43 | R | Overijssel | 2&3 | 1 | 158 | 1 | 4 | not present | not present |  |
| VBS 44 | E2 | Utrecht | 2 | - | - | 1 | 2 | - | - |  |
| VBS 45 | E1 | Utrecht | 2&3 | - | 78 | 4 | 9 | - | - |  |
| VBS 46 | R & P | Zuid-Holland | 3 | 1 | 90 | 1 | 3 | not present | not present |  |
| VBS 47 | R. | Noord-Holland | 2 | 1 | 48 | 1 | 3 | not present | present |  |
| VBS 48 | E2 | Noord-Holland | 1 | - | 140 | 1 | 2 | - | - |  |
| VBS 49 | R | Friesland | 2&3 | - | - | 1 | 3 | - | - |  |
| VBS 50 | E3 | Zuid-Holland | 1 | 1 | 36 | 2 | 2 | not present | present |  |
| VBS 51 | R | Zuid-Holland | 1 | 1 | 42 | 6 | 2 | not present | not present |  |
| VBS 52 | R | Friesland | 1 | 1 | 42 | 6 | 1 | not present | not present |  |
| VBS 53 | R | Overijssel | 1 | - | - | 1 | 6 | - | - |  |
| VBS 54 | R & E1 | Zuid-Holland | 1&2&3 | - | 142 | 2 | 6 | - | - |  |
| VBS 55 | R | Noord-Holland | 2 | - | 75 | 2 | 4 | - | - |  |
| VBS 56 | R & E1 | Utrecht | 2 | - | 80 | 3 | 4 | - | - |  |
| VBS 57 | R | Utrecht | 1 | 1 | 400 | 5 | 7 | not present | present |  |
| VBS 58 | R | Zuid-Holland | 1 | 1 | 150 | 5 | 2 | not present | present |  |

- = no information

*1 Curriculum:
R = Entrepreneurship is offered in the regular hours of Economics, Business Economics, Economics and Entrepreneurship or other courses.
E1 = Entrepreneurship is offered in one extra lesson per week.
E2 = Entrepreneurship is offered in two extra lessons per week.
E3 = Entrepreneurship is offered in three extra lessons per week.
D1 = Entrepreneurship is offered in one half day (4 lessons) per week (10/90 learning).
D2 = Entrepreneurship is offered in one day (8 lessons) per week (20/80 learning).
EX: Extra entrepreneurship hours per year + number
P= Project weeks

*2 Level:
Level 1 = preparatory secondary vocational education

Level 2 = senior general secondary school

Level 3 = pre-university education

*3 Mission and vision:

This refers to a separate mission and vision with regard to entrepreneurial learning. So not the general mission and vision of the school.

*4 Learning objectives:

This refers to the learning objectives of the entire program offered. So not on the learning objectives of every purchased program. Such as the accounting module, in which learning objectives are included in the method.
