# Supplementary material for: Entrepreneurship programs and their underlying pedagogy in secondary education in the Netherlands
Source: Entrep Educ. 2022 Oct 12;5(3):261–87. doi: 10.1007/s41959-022-00078-8 (PMC13295173; doi:10.1007/s41959-022-00078-8)
Supplement: Supplementary file 2 — Supplementary file2 (DOCX 24 kb) [file 41959_2022_78_MOESM2_ESM.docx]

Appendix 2: The way of scoring for each design principle based on a threefold scale: low/simple, hybrid, and high/complex.

| **11 design principles** | **Low(er) levels of complexity and uncertainty** | **Hybrid** | **High(er) levels of complexity and uncertainty** |
| --- | --- | --- | --- |
| **1. The entrepreneurial process** | | | |
| Method | Schools developed an entrepreneurship program based on various purchased and self-designed programs. We scored entrepreneurship programs low among schools that offered only causally focused programs, such as accounting, marketing, and other theoretically designed assignments. | We gave hybrid scores to those entrepreneurship programs that included at least one impact-oriented program with room for students' entrepreneurial skills, ideas, and interests. | We scored entrepreneurship programs highly, which contained many impact-oriented programs. Unfortunately, we did not encounter examples of these schools in our sample. |
| Level of autonomy | We saw that schools that scored low on the first design principle often scored low here, and vice versa, because causality-based instruction was structured and teacher-led. Thus, schools where the teacher had a central role in teaching entrepreneurship, scored low. | We scored programs in schools hybrid if students were offered more autonomy within one or a few projects (mini-companies) in pre-structured frameworks. Teachers themselves felt more like coaches than knowledge transmitters as a result. | We scored programs in schools highly, in which students were given autonomy in multiple assignments. Unfortunately, we did not encounter examples of these schools in our sample. |
| Room to maneuver | We scored programs low in schools that gave students feedback only on assessments and grades. Students here were only given the space to try out and reflect in a safe setting. Examples of this type of causally oriented program were: accounting, marketing, and other theoretical assignments. | We scored programs hybrid to schools, giving students process-oriented feedback on one or a few assignments/projects. Here, students were given the space to test and reflect on it. Also, within these projects, these schools gave students space to make mistakes in specific frameworks and learn from their own mistakes. | We scored programs high in schools that offered students short cycles of prototyping-testing-reflecting in multiple assignments/projects, where making mistakes and time pressure was part of the process. |
| **2. The task** | | | |
| The complexity of the cases students face | Entrepreneurship programs were scored low if they were primarily theoretical. Also, we scored constructivist programs in which only buying and selling of products took place (AliExpress) low because the complexity of these assignments was low. | Entrepreneurship programs were scored hybrid if innovation played a more prominent role within the constructivist assignments. An example is an electric bicycle that allows one to charge a phone on the go. | The entrepreneurship programs were scored high if the innovation content within the constructivist assignments was high. Assignments in which students were challenged to develop something new for the world, for example, to solve social problems. |
| Nature of the value creation | Entrepreneurship programs in which students had to work on relatively simple business ideas, focusing only on economic value creation, scored low. | In entrepreneurship programs, in addition to economic value creation, attention was paid indirectly (not as a requirement of the assignment, but is rewarded if they work with it) to social, cultural, or environmental value creation; we scored hybrid. | In entrepreneurship programs with multiple value creation (economic, social, cultural, and environmental), we scored high. |
| Knowledge creation process | We scored programs in entrepreneurship low when the focus in the programs was not on using knowledge but on learning by doing (releasing knowledge in a creative and out-of-the-box process). | We scored programs in entrepreneurship hybrid when in addition to being creative and providing an out-of-the-box learning process (learning by doing), space was also provided to indirectly build on the knowledge that is ultimately crucial for innovative entrepreneurship. | We scored entrepreneurship programs high when, in addition to being creative and offering an out-of-the-box learning process (learning by doing), space was also offered to build directly on the knowledge that is ultimately crucial for innovative entrepreneurship. |
| Impact of the result | Entrepreneurship programs in which the impact of the value creation process was on the student or teacher scored low. | Entrepreneurship programs in which the value creation was at the local level scored hybrid/high, depending on the nature of the project or assignment. | Entrepreneurship programs in which the value creation took place at the (inter)national level scored high, depending on the nature of the project or assignment. |
| **3. The context and relationships** | | | |
| Context/ environment | We scored low in entrepreneurship programs where the value creation process took place at the local level. | In entrepreneurship programs where the value creation process took place within one or a few components on a national level, we scored hybrid. | We scored high in entrepreneurship programs where the value creation process took place on an (inter)national level. |
| Cooperation | Entrepreneurship programs often required students to work individually or in pairs scored us low. | We scored hybrid in entrepreneurship programs that often required students to work in groups (often in groups of two to six students) but not across class or grade boundaries. | In entrepreneurship programs where collaboration also took place in multidisciplinary or interdisciplinary groups or teams or even in transdisciplinary teams involving stakeholders from outside the school, we scored high. |
| The role of external stakeholders | We scored low in entrepreneurship programs where the role of external stakeholders was low. | In entrepreneurship programs where external coaches or entrepreneurs played a role, we scored hybrid. | We scored high in entrepreneurship programs that required a strong network and a high intensity of coordination. |
| Role models | Entrepreneurship programs that used role models to inspire and motivate students scored low. | In entrepreneurship programs in which role models were used to coach students in addition to inspiring and motivating students, we scored hybrid. | We scored high in entrepreneurship programs in which role models were used to work on students' identity formation and personal growth in addition to inspiring, motivating, and coaching students. |
