# Supplementary material for: Entrepreneurship programs and their underlying pedagogy in secondary education in the Netherlands
Source: Entrep Educ. 2022 Oct 12;5(3):261–87. doi: 10.1007/s41959-022-00078-8 (PMC13295173; doi:10.1007/s41959-022-00078-8)
Supplement: Supplementary file 3 — Supplementary file3 (DOCX 34 kb) [file 41959_2022_78_MOESM3_ESM.docx]

Appendix 3: The different parts of the upper secondary business schools programs.
Entrepreneurship programs in secondary education, Number of study hours, The level at which it is offered, and a Brief Explanation of the programs.

| **Programs in upper secondary entrepreneurship education** | **Purchased, subsidized/free or designed** | **Pedagogy** | **Number of study hours** | **The level at which it is offered** | **Explanation** |
| --- | --- | --- | --- | --- | --- |
| **Accountancy** |  |  |  |  |  |
| Basic Knowledge Accounting (BKB) | purchased | Traditional, ”about” entrepreneurship  Teacher centered | 40 hours | pre-vocational secondary education / senior general secondary education/pre-university education | The Association Diploma Basic Knowledge Accounting (BKB) offers the student knowledge and insight into accounting. |
| EBC*L, level A | purchased | Traditional, ”about” entrepreneurship  Teacher centered | 30 hours | senior general secondary education/pre-university education | The EBC*L (level A) course offers the student knowledge about the topics: company form and legislation, costs and prices, financial administration, company objectives, and key figures. |
| **Marketing** |  |  |  |  |  |
| NIMA Basic Knowledge Marketing | purchased | Traditional, ”about” entrepreneurship  Teacher centered | 30 hours | senior general secondary education/pre-university education | The marketing Basic Diploma offers the student knowledge and insight into Marketing and Entrepreneurship. Topics include product and pricing policy, distribution, consumer behavior, and the latest insights and techniques within online marketing. |
| **Student companies / business plans** |  |  |  |  |  |
| Gofuture Business | purchased | Constructive,  ”for” entrepreneurship  Teaching-centered | varies  80 hours | pre-vocational secondary education / senior general secondary education/pre-university education | The Gofuture Business program includes a computer simulation. Students learn to set up their own company or organize an event using the Business Model Canvas. Each school is free to give its interpretation and twist to the program. |
| Gofuture | purchased | Constructive,  ”for” entrepreneurship  Teaching-centered | varies  60 hours | pre-vocational secondary education | In the Gofuture program for pre-vocational secondary education, students learn to set up their own (fictional) company using the 4 P's (product, price, place, and promotion). Each school is free to give its interpretation and twist to the program. The students learn this using a simulation on the computer. |
| Junior Company | purchased | Constructive,  ”for” entrepreneurship  Teaching-centered | varies  40-120 hours | all levels | Junior Company is a program of Jong Ondernemen that provides insight into upper secondary students in secondary education. Students set up their own business in the program and run it for a school year/period. They allocate functions, determine the target group, brainstorm about their product, write a business plan, sell shares and keep financial records. |
| Own developed versions of Junior Company | developed | Constructive,  ”for” entrepreneurship  Teaching-centered | varies | all levels | Schools have developed their version of the Junior Company project (students set up their own business in the program and run it for a school year/period) because they can then deal with the content more flexibly and are cheaper. |
| Webshop at school | purchased | Constructive,  ”for” entrepreneurship  Teaching-centered | varies 10-15 hours | pre-vocational secondary education | At Webshop at School, lower general secondary education students learn everything about designing a webshop. This teaching package is offered by Jong Ondernemen, is intended for lower secondary education, but is also used in upper secondary education. This teaching package focuses on entrepreneurial and digital skills when students are setting up their own webshop. |
| Eurobizz company: Entrepreneurship at School | purchased | Constructive,  ”for” entrepreneurship  Teaching-centered | varies 24-88 hours | all levels | Within the Entrepreneurship at School program, students are challenged in two phases (introduction to entrepreneurship and implementation) to design a product/concept and fully develop it in 12 practical assignments and at least a company visit. The organization arranges everything itself, including trainers who direct the teachers. |
| Qredits: Own Boss | purchased | Constructive,  ”for” entrepreneurship  Teaching-centered | Varies  20-30 hours | senior general secondary education/pre-university education | Qretis' Own Boss program is an e-learning program for senior general secondary education and pre-university education. Students will be exposed to the real business world through the program: they will discuss a business case with a Qredits loan officer and then start writing their own business plans and eventually pitching it in front of a jury. |
| Qredits: Own Boss as elective course | purchased | Constructive,  ”for” entrepreneurship  Teaching-centered | 100 hours | pre-vocational secondary education | The Own Boss Educational Program for the lower general secondary education can be used as an elective course 'Entrepreneurship.' Students work within the e-learning program on developing a business plan. |
| **Skill developments** |  |  |  |  |  |
| Presentation skills / manners module | designed | Constructive,  ”for” entrepreneurship  Teaching-centered | 10 hours | all levels | The students learn in the module presentation skills/manners about different presentation techniques and manners. |
| Excel module | designed | Traditional, ”about” entrepreneurship  Teacher centered | 10 hours | senior general secondary education/pre-university education | In the Excel module, the students learn how to make business economic calculations in Excel. |
| **Projects** |  |  |  |  |  |
| Projects with further education (different versions) | designed | Constructive,  ”for” and ”through”  Teaching-centered / Learner centered | varies | senior general secondary education/pre-university education | Senior general secondary education and pre-university education schools have projects running with universities. Secondary school students attend university lectures and work in groups on projects/cases (on paper) with university students. Often there is a competitive element in this. The groups compete against each other and from which a winner emerges. |
| **Social/ environmental enterprise** |  |  |  |  |  |
| Day for Change | purchased | Constructive,  ”for” entrepreneurship  Teaching-centered | 40 – 60 hours | all levels | Day for Change is an organization that offers guest lectures, lesson letters, workshops, and teaching materials. The teaching material is for a project in which students set up their own sustainable business: the Day for Change Action. The proceeds of their business are donated to charity. |
| Global Goals | designed | Constructive,  ”for” entrepreneurship  Teaching-centered |  |  | Students must design a product or service based on at least one Global Goal. Based on this, they set up a company and write a business plan. |
| Social Innovation Relay van Jong Ondernemen | subsidized | Constructive,  ”for” entrepreneurship  Teaching-centered |  |  | Students have to tackle a social or environmental problem by working in a team to develop a social and innovative concept. The best 20 teams (national) will advance to the 2nd round and receive a coach to develop their concept into a feasible plan further. The best team in the Netherlands advances to the worldwide competition. |
| **Reflection / test** |  |  |  |  |  |
| E-Scan | purchased | Constructive,  ”for” entrepreneurship  Teaching-centered | 5 hours | all levels | The E-scan is an entrepreneurial test to uncover students' entrepreneurial potential, traits, and skills in 15 minutes. This tool is used at the beginning of group assignments to form groups and provide insight into the qualities of the student as an entrepreneur. Website: https://entrepreneurscan.com/nl/ondernemerstest/ |
| Reflection | designed | Constructive,  ”for” entrepreneurship  Teaching-centered | 5 hours | all levels | Students take various entrepreneurship tests within the reflection assignment and question their environment to map out their strengths and weaknesses. Based on this, they develop their learning objectives. Creating a Curriculum Vitae and applying for jobs is also part of this. |
| **Gamification** |  |  |  |  |  |
| Bizzgames | purchased | Constructive,  ”for” entrepreneurship  Teaching-centered | varies  8-40 hours | all levels | Bizzgames is an organization that offers management games for upper secondary education. These management games can be used in secondary education in business economics, Economics, Administration, Trade, sales, and consumer. The form in which the games are offered varies from a practical assignment, project, integrated test, sector assignment, or teach entrepreneurship. |
| Econasium: Plaza Challenge School | purchased | Constructive,  ”for” entrepreneurship  Teaching-centered | varies  6-15 hours | pre-university education | Plaza Challenge School is an economically oriented online management game for upper secondary school students. In Plaza Challenge, pupils experience what it is like to start a shop themselves in a virtual shopping center where they compete with and against each other. |
| **Financial literacy** |  |  |  |  |  |
| MoneyWays | subsidized | Constructive,  ”for” entrepreneurship  Teaching-centered | varies 8-15 hours | all levels | MoneyWays is a national project about dealing with money for secondary education about money, debt, and poverty. The program consists of three workshops, which peer educators give. In addition, various teaching modules are made available, which teachers can use. |
| TELMA: No credit, no game.. | subsidized | Constructive,  ”for” entrepreneurship  Teaching-centered | varies 8-15 hours | all levels | No Credit, Game Over!® is a city game that moves young people around a socially relevant theme: "debt." Using digital media, students must make their way through the city in an attempt to pay off their character's debts. In the end, it is discussed in order to evaluate the behavior and decisions of students. |
| **Language** |  |  |  |  |  |
| Cambridge Business English | purchased | Traditional, ”about” entrepreneurship  Teacher centered | varies 40-60 hours | senior general secondary education/pre-university education | Students are allowed to qualify for the Cambridge Business English certificate in addition to the English subject. The students are challenged to develop their English language skills further to communicate confidently in an international workplace/company. The Cambridge Business diploma is also internationally recognized. |
| **Activities** |  |  |  |  |  |
| Guest lessons | free | Constructive,  ”for” entrepreneurship  Teaching-centered | varies  5-10 hours | all levels | At least two guest speakers from different industries are invited to talk about their experiences and work as an entrepreneur. |
| Company visits | free | Constructive,  ”for” entrepreneurship  Teaching-centered | 5 hours | all levels | Company visits to companies, distribution centers, and factories are planned once or twice a year to give students a glimpse of the field. |
